# Study on the Controllable Preparation of Nd^3+^ Doped in Fe_3_O_4_ Nanoparticles for Magnetic Protective Fabrics

**DOI:** 10.3390/molecules28073175

**Published:** 2023-04-03

**Authors:** Xiaolei Song, Congzhu Xu, Wendong Yao, Jieyun Wen, Qufu Wei, Yonggui Li, Xinqun Feng

**Affiliations:** 1 Fujian Key Laboratory of Novel Functional Textile Fibers and Materials, Minjiang University, Fuzhou 350108, China; 2 Faculty of Clothing and Design, Minjiang University, Fuzhou 350108, China; 3 College of Textile and Clothing, Jiangnan University, Wuxi 214122, China; 4 College of Fashion and Design, Donghua University, Shanghai 201620, China

**Keywords:** NdFe_2_O_4_ nanoparticles, grafting, cotton fabric, magnetic protective

## Abstract

Magnetic protective fabrics with fine wearability and great protective properties are highly desirable for aerospace, national defense, and wearable protective applications. The study of the controllable preparation method of Nd^3+^ doped in Fe_3_O_4_ nanoparticles with supposed magnetic properties remains a challenge. The characterization of the microstructure, elemental composition, and magnetic properties of NdFe_2_O_4_ nanoparticles was verified. Then, the surface of NdFe_2_O_4_ was treated with glyceric acid to provide sufficient –OH. Subsequently, the connection of the nanoparticle by the succinimide group was studied and then grafted onto cotton fabrics as its bridging effect. The optimal loading rate of the functional fabrics with nanoparticles of an average size of 230 nm was 1.37% after a 25% alkali pretreatment. The color fatness to rubbing results showed better stability after washing and drying. The corresponding hysteresis loop indicated that the functional fabrics exhibited typical magnetism behavior with a closed “S” shape and a magnetic saturation value of 17.61 emu.g^−1^ with a particle size of 230 nm. However, the magnetic saturation value of the cotton fabric of 90 nm was just 4.89 emu.g^−1^, exhibiting controllable preparation for the aimed electromagnetic properties and great potential in radiation protective fields. The electrochemical properties of the functional fabrics exhibited extremely weak electrical conductivity caused by the movement of the magnetic dipole derived from the NdFe_2_O_4_ nanoparticles.

## 1. Introduction

The rapid development of electronic information technology, military science, and intelligent manufacturing technology in the 21st century has provided many conveniences and high−quality services [1,2]. However, the increasing electromagnetic radiation pollution woul damage the environmental climate, the smooth operation of precision instruments, and eventually human health, especially with the promotion of 5G networks and an increase in related products. These concerns have raised great attention in both scientific and industrial circles [3,4,5,6]. Since the electromagnetic characteristics of functional fabrics involve dielectric and magnetic loss, it is necessary to give much consideration to studies on electromagnetic wave absorption and recurrent reflex design [7]. Therefore, urgently developing a novel electromagnetic protective lightweight fabric with high−performance has become a research hot-spot in recent decades.

Recently, multicomponent dielectric and magnetic loss materials with honeycomb porous, core−shell, hollow, multilayer, or snowflake structures have been developed as effective electromagnetic wave radiation products [8,9,10]. The dielectric and magnetic loss materials, as well as the microstructures, can endow the micro/nano functional units with excellent impedance matching and synergetic electromagnetic losses. Xu et al. [11] reported on high−performance electromagnetic interference shielding graphene materials with honeycomb porous structures, simultaneously with ultralow density, excellent flexibility, and good mechanical properties using a laser scribing technology. Zhang et al. [12] studied a multilayer structure of the growth of Fe_3_O_4_ nanoparticles onto the Ti_3_C_2_T_x_ MXene surface and interlayer nanocomposites for enhancing microwave absorption properties. Lei et al. [13] revealed a simple freeze−drying route for designing a thermoplastic polyurethane composite with a snowflake structure, consisting of silver fractal dendrites, carbon nanotubes, and thermoplastic polyurethane for final studies on electromagnetic interference shielding properties.

High magnetic permeability is the primary factor determining the magnetic shielding properties of a material, which is favorable for a multi-reflection loss mechanism [14,15]. Among them, monodispersed Fe_3_O_4_ and Fe_2_O_3_ nanomaterials with superparamagnetic properties have been prepared for magnetic protective and medical drug conduction materials using ethanol and aqueous media. However, pure Fe_3_O_4_ nanoparticles (NPs) show high a saturation magnetization (80 emu.g^−1^) and a high coercive force (55 Oe), and they are widely used in magnetic protective materials and medical fields [16]. Many doping plans in the Fe_3_O_4_ crystal structure, such as Ni, La, Co, and Mn, have attracted much attention for their usability in magnetization and functional stability. Doping spherical NPs of M (M = Ni, Cu, Co, Zn, Au) into the Fe_3_O_4_ crystal can efficiently improve the magnetic performances of naked Fe_3_O_4_ at different degrees [17]. For instance, CoFe_2_O_4_@MgFe_2_O_4_ NPs have excellent magnetic saturation properties based on external spectroscopy in the magnetic hyperthermia field [18]. Neodymium exhibits excellent reactive properties, thermal stability, and effective paramagnetism when Nd^3+^ is doped into Fe_3_O_4_ during the crystal formation process, and the size, shape, and magnetic properties of the obtained particles can be improved [19]. Considering the loss of dielectric, magnetic, and reflection properties during the development of protective products, the conductivity and permeability performance of the functional materials must be revealed. At low frequencies, the magnetic losses are dominated by high permeability materials, which could be caused saturation with an increase in magnetic field strength. However, dielectric loss was mainly contributed to by fine conductivity materials at high frequencies. Ji and his team members [20] successfully prepared multilayer energy loss materials for clarifying the mechanism of compound electromagnetic loss with a thick and heavy structure.

However, considering the ubiquitous difficulty of easily aggregating, surface modification is required for Fe_3_O_4_−based NPs to improve their dispersion and stability. For instance, a fine composite of molybdenum disulfide@polypyrrole decorated with modified doping Fe_3_O_4_ NPs was shown to improve stability in the sensing field as an electromagnetic matching material. The core−shell structure of Fe_3_O_4_@SiO_2_ NPs with fine dispersion in thermosensitive poly (N−isopropylacrylamide) and luminescent lanthanide polyoxometalates was previously described for wearable flexibility materials [21,22,23].

According to the Schelkunoffs transmission theory [24,25], the electromagnetic interference of shielding materials with SE reflection (SE_R_) and absorption (SE_A_), is mainly correlated with their electrical conductivity at high frequencies and magnetic permeability at low frequencies. However, reflection occurs once the incident waves reach the surface of the shielding materials, which is undesirable due to the risk of secondary radiation contamination [26]. Therefore, the loss in the process of multi reflection and absorption, which is attenuated by the movement and transferring of electric and magnetic dipoles in the shielding products under certain electric and magnetic fields, is greatly dependent on the materials’ magnetic permeability, electrical conductivity, and thickness [27]. Xu et al. [28] studied a waterborne polyurethane composite film with multilayer rGO@Fe_3_O_4_ structures, exhibiting an excellent electromagnetic shielding and a low reflection. A similar approach was taken by Duan and his team members [29] for designing an asymmetric conductive polymer composite foam with extremely low reflection characteristics as a shielding material.

However, the X−band frequency range, from 8.2 to 12.4 GHz, is widely used in radar detection and camouflage applications, and it is applicable to the flexible sample test via the rectangular waveguide method [30,31]. To obtain efficient magnetic protective fabrics, this work studied the effects of particle size on the magnetic performance of functional fabrics in the X−band. Firstly, a controllable-sized NdFe_2_O_4_ NP was developed using the solvent thermal synthesis method with a microwave synthesizer. The as−prepared NdFe_2_O_4_ NPs with average particle sizes of 90 nm and 230 nm were respectively studied for related structures and magnetic properties. Then, the functional groups on the surface of the NPs after modification were connected with rich −OH on cotton fabrics using a bridging agent for durable interface graft bonding. By using the above method, NdFe_2_O_4_ NPs with spherical shapes, fine dispersions, magnetic properties, and final functional fabrics with magnetic protective properties were developed. The structure and relevant properties of the as−prepared NPs and final fabrics were systematically characterized and analyzed.

## 2. Results and Discussion

### 2.1. Illustration of the Preparation Process

**Figure 1:**
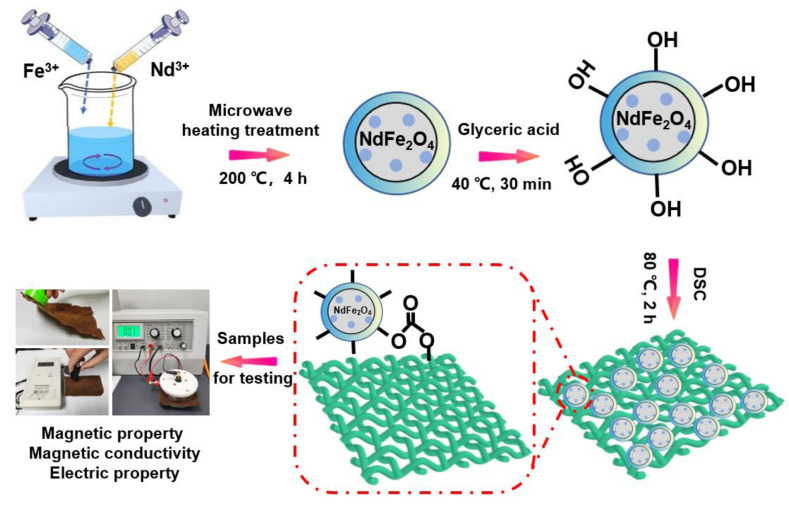
Illustration of the preparation process of the reliable magnetic protective fabrics.

The detailed preparation process of the functional fabrics is diagrammatically exhibited in Figure 1, which includes the solvothermal synthesis and surface modification for reliable magnetic protective fabrics. First, a typical synthesis reaction was successfully performed to prepare controllable NdFe_2_O_4_ NPs. Glyceric acid was then used to improve the reaction capacity of NPs for further interface bonding. The structural formula of glyceric acid contains one carboxyl group and two hydroxyl groups. The carboxyl group of glyceric acid connects with the hydroxyl group on the surface of the particles through dehydration and condensation to form an ester group, thus obtaining the hydroxylation product of the nanoparticles. The cotton fabrics were fabricated with a fine appearance and performance through a weaving machine in our laboratory, and the samples were treated with a NaOH solution at a concentration of 25% to improve the surface activity for rich hydrophilic groups. Subsequently, the proper number of moles of DSC were added into the NdFe_2_O_4_ NPs dispersion, which acted as a bridge agent between the final fabrics and the NPs. The carbonated group on one side of the succinamide combined with the hydroxyl group on the surface of the particle through a dehydration condensation reaction to form an ester bond at a low temperature (40 °C). Meanwhile, the oxygen on the other side of the succinamide carbonate combined with the hydrogen proton in the hydroxyl group on the surface of the cotton fiber at a high temperature (80 °C). Then, the reliable cotton fabrics with durable magnetic protective properties were obtained.

### 2.2. Characteristics and Magnetic Properties of NdFe_2_O_4_ NPs

**Figure 2:**
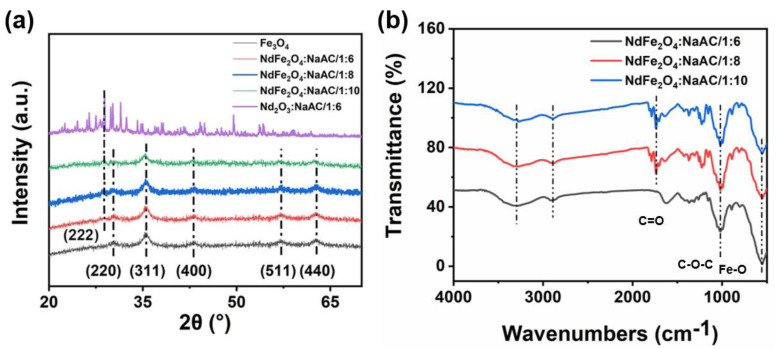
(**a**) XRD patterns, and (**b**) FT−IR patterns of Fe_3_O_4_ and NdFe_2_O_4_ NPs.

The crystal forms of Fe_3_O_4_, Nd_2_O_3_, and NdFe_2_O_4_ NPs with different mole ratios of NaAC were detected though XRD, as shown in Figure 2a. The typical XRD pattern peaks of Fe_3_O_4_ were observed at 30.1°, 35.4°, 43.0°, 56.9°, and 62.5°, corresponding to the (220), (311), (400), (511), and (440) planes, respectively, which were consistent with the Joint Committee on Powder Diffraction Standards of Fe_3_O_4_ (JCPDS Card no: 65−3107) [32]. Furthermore, the typical lattice plane (222) of Nd_2_O_3_ at 27.891° (JCPDS Card no: 65−3187) appeared in the corresponding doping curves. Thus, the solvent thermal synthesis of Nd doping in Fe_3_O_4_ was successful in different mole ratios of NaAC, which supports previous reports. As the NaAC concentration increased from 1;6 to 1;10, the crystallite sizes were 13.2 dm, 13.6 nm, and 14.5 nm, respectively. The trend of the data was not obvious at this stage. Figure 2b shows a distinct absorption peak located at 582 cm^−1^ in the patterns, which could be attributed to the vibration of the Fe−O group that was obtained from the NdFe_2_O_4_ crystal structure [33]. Furthermore, the common peaks at 1046 and 1627 cm^−1^ corresponded to the hierarchical ether group (−C−O−C−) and carbonyl group (C=O), respectively [34]. However, the carbonyl group peaks among the three samples were similar because of the oxidation of hydroxy from PEG in the reducing reaction of partial Fe^3+^ to Fe^2+^ [35]. Considering the influence of the small size effect, some adsorption peaks showed a slight shift, as shown in the spectrum. However, the characteristic peak of the Nd−O group was located at 345 cm^−1^, which was out of this test range and further analyzed by XPS.

**Figure 3:**
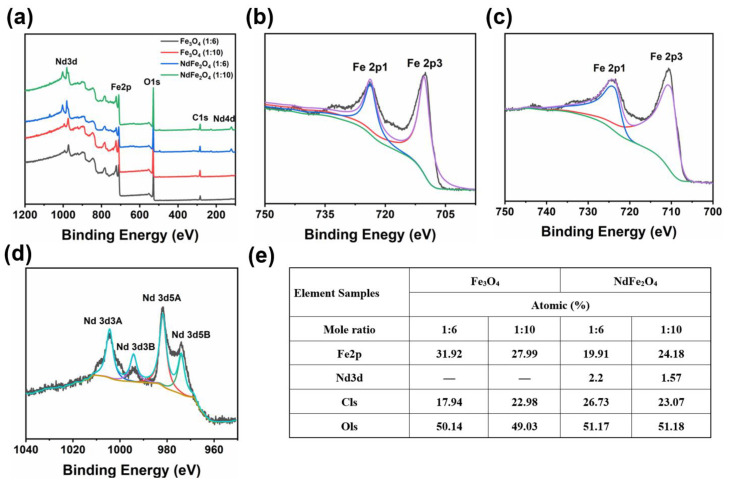
XPS survey spectra and high-resolution spectra of Fe_3_O_4_ and NdFe_2_O_4_ NPs. (**a**) XPS full spectrum; (**b**) Fe spectrum of Fe_3_O_4_ NPs; (**c**) Fe spectrum of NdFe_2_O_4_ NPs; (**d**) Nd spectrum of NdFe_2_O_4_ NPs; (**e**) Atomic percent of the as-prepared NPs.

The elemental composition and the corresponding chemical binding energies of each element were investigated by examining the Fe_3_O_4_ and NdFe_2_O_4_ NPs at different mole ratios of NaAC via XPS in Figure 3. The survey scan patterns (Figure 3a) showed the binding energies of C and O in all the curves, which were located at 286.4 and 529.8 eV, respectively, while Nd 4d was only observed in the curves of NdFe_2_O_4_. The high−resolution peak of Fe 2p and Nd 3d for Fe_3_O_4_ and NdFe_2_O_4_ NPs were further confirmed in Figure 3b−d. Two obvious peaks were centered at 710.1 and 724.2 eV, which corresponded to Fe 2p1 and Fe 2p3 derived from Fe−O bonds, respectively [36]. In addition, the Nd 3d narrow scan peaks were detected at 974.12, 981.6, 994.4, and 1,003.5 eV, which could be assigned to Nd 3d5A, Nd 3d5B, Nd 3d3A, and Nd 3d3B, respectively [37]. The atomic percent data shown in Figure 3e indicated the elemental composition of the as-prepared NPs, exhibiting the successful doping of Nd in the Fe_3_O_4_ crystal structure without changing the valence state of the Fe. The XPS analysis above provides favorable evidence for the successful preparation and structural confirmation of the Nd doped in Fe_3_O_4_ crystal, which were consistent with the above XRD observations.

**Figure 4:**
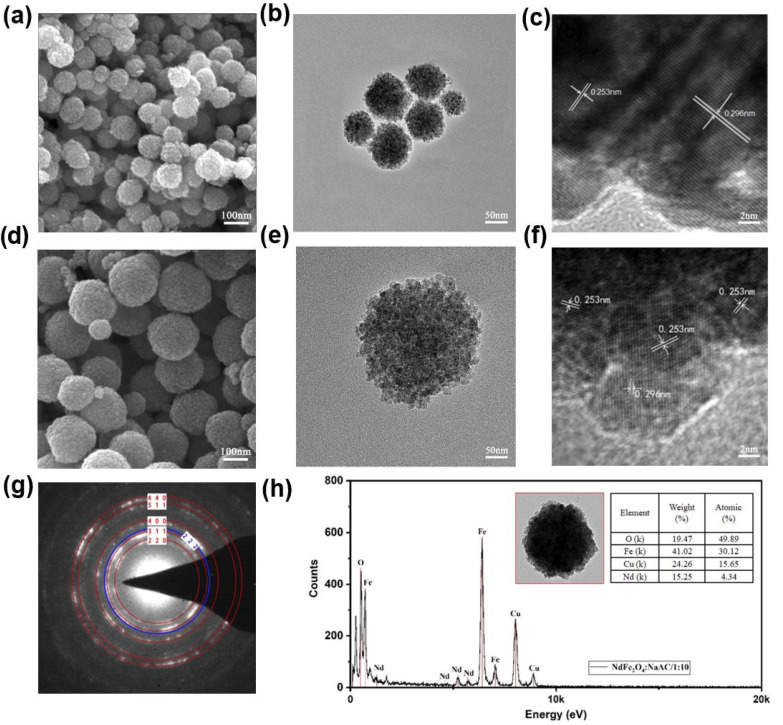
Characterization of NdFe_2_O_4_ NPs. (**a**) SEM image, (**b**) TEM image, and (**c**) HR−TEM image of NdFe_2_O_4_ NPs at 1:6 NaAC; (**d**) SEM image, (**e**) TEM image, (**f**) HR−TEM image, (**g**) electron diffraction, and (**h**) EDS pattern of NdFe_2_O_4_ NPs at 1:10 NaAC.

Apart from the element and crystal analysis of the sample, the surface morphology was also investigated. Figure 4 presents the TEM and SEM images of Fe_3_O_4_ and NdFe_2_O_4_ NPs prepared under different parameters together with the high-magnification lattice fringes and electron diffraction results. The as−prepared NdFe_2_O_4_ NPs had regular spherical structures at major particle sizes of 70−110 nm (Figure 4a,b) in 1:6 NaAc. However, larger NPs were obtained with a major size range of 200−270 nm (Figure 4d,e) in 1:10 NaAC, which can be attributed to the different reduction ability in different mole ratios of the Fe source to NaAC for the formation of the magnetic crystal [38]. Subsequently, the lattice fringe spacing showed common fringe spacings of 0.253 and 0.296 nm, corresponding to the (311) and (220) lattice planes from JCPDS Card no: 65−3107, respectively (Figure 4d,f). The electron diffraction pattern (Figure 4g) of the product displayed evident (220), (311), (400), (511), and (440) crystal plane electron diffraction signals, which belonged to the Fe_3_O_4_ crustal [39]. In addition, the blue electron diffraction signal of the (222) crystal plane coincided with the Nd product of JCPDS Card no: 65−3187, which was consistent with the XRD and XPS results of the above representations [40]. The body elements of NdFe_2_O_4_ were Fe, O, Cu, and some Nd in an atomic percentage (Figure 4h). In the measured area, the atomic percentage of Cu was associated with the test condition of the Cu net for the weight tray. The atomic percentage of Nd was only 4.34%, whereas that of Fe was 30.12% and that of O was 49.89%. This result can be attributed to the larger ionic radius of Nd^3+^ than that of Fe^2+^ [41], which reduced the crystallization capacity of Nd in Fe_3_O_4_. Thus, the production of Nd was successfully doped into the Fe_3_O_4_ crystal structure, which was consistent with the above XPS observations.

**Figure 5:**
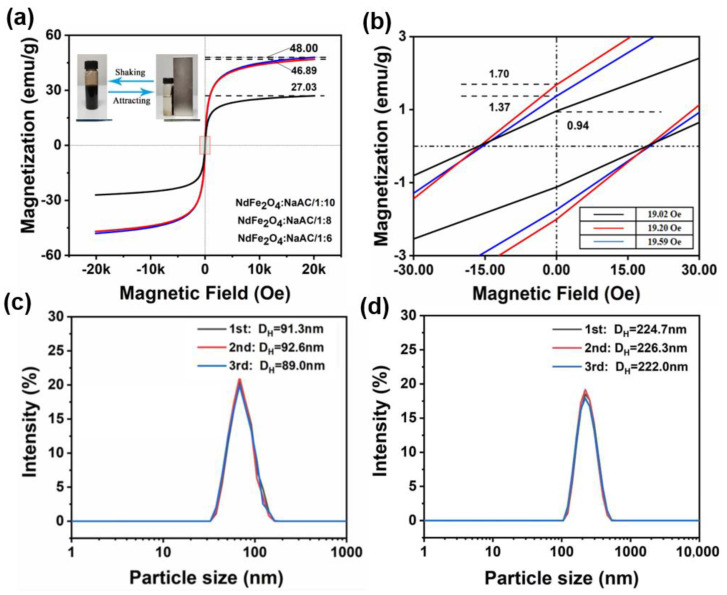
Magnetic performance of NdFe_2_O_4_ NPs. (**a**) M−H curves; (**b**) enlarged M−H curves; DLS data at 1:6 (**c**), and 1:10 (**d**) of NdFe_2_O_4_ NPs.

The magnetization performances of the obtained NdFe_2_O_4_ NPs were measured using VSM, and their hysteresis loops with closed “S” shapes are shown in Figure 5a. The saturation magnetization values of the samples were 27.03, 46.89, and 48.00 emu.g^−1^, indicating the differences in magnetization capacity [42]. Combined with the corresponding TEM results, NPs with a small particle size (at a mole ratio of 1:6) showed low saturation magnetization value of 27.03 emu.g^−1^, but it was close to 48 emu.g^−1^ for samples prepared at higher exposure times to NaAC, possibly because of the effect of particle size in different mole ratio of the iron source to NaAC. Thus, the particle size plays a key role in determining magnetism properties [43]. As shown in the inset picture, the prepared NPs could realize the rapid switch between good dispersion and absorption on the wall with and without the action of the magnet [44]. Thus, the as-prepared NPs were well-dispersed in water at room temperature, thereby promoting their medical and biological protective applications. The residual magnetization value and coercive force obtained from the enlarged curves (Figure 5b) are relatively close, which was beneficial for enhancing the following functional fabrics’ electromagnetic protective performances for the prospective applications [45].

Particle size analysis was carried out to measure the DLS distribution curves of the NPs at a 1:6 and 1:10 ratio for three times (Figure 5c,d). The tested particle size was mainly distributed at approximately 90 and 230 nm, respectively, which was generally consistent with the SEM and TEM results [46]. However, the polymer dispersity index values of the obtained NdFe_2_O_4_ NPs were between 0.071, 0.111, and 0.121, 0.183, respectively. This finding indicates a good shape and controllable particle size, and a slightly larger dispersion rate in 1:10 samples than that of the 1:6 samples, which support the above VSM results.

### 2.3. Characteristics and Magnetic protective of the Functional Fabrics

**Figure 6:**
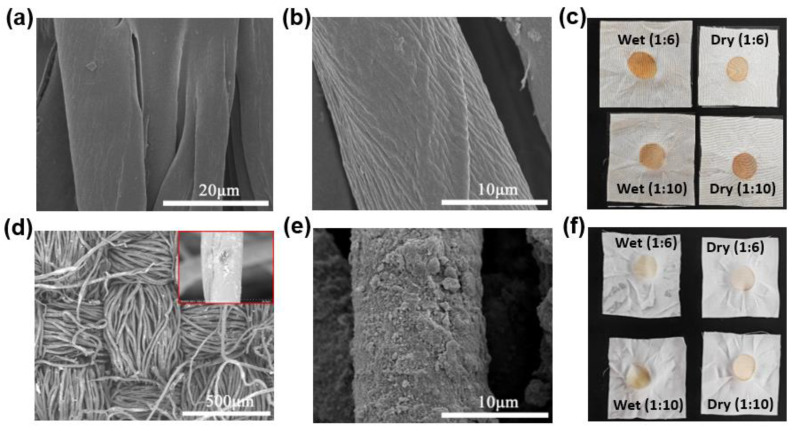
Characterization of the functional fabrics. SEM images of (**a**) before and (**b**) after alkali treatment; SEM images of the grafting fabrics (**d**) before and (**e**) after washing for three times; color fatness to rubbing results of (**c**) before and (**f**) after washing the fabrics for three times.

Glyceric acid and DSC were selected for the grafting of NdFe_2_O_4_ NPs onto cotton fabrics for durable wearability. The best load rate of the functional fabrics was 1.37% at room temperature, which can be attributed to the best water absorption of cotton fabrics following a 25% alkali treatment. The SEM images of the cotton fabrics before and after alkali treatment at a concentration of 25% are shown in Figure 6a,b. The surface of the fiber was quite smooth, a rough and etching shape of the cellulose fiber was obtained after the alkali treatment, and the surface activity was improved, which was useful for the following grafting of NPs. The sample grafted with a proper amount of the prepared NdFe_2_O_4_ NPs is shown in Figure 6d,e. The as−grafted fabric possessed a uniformly thin layer of nanosheet structure and showed an adequate grafting response to cellulose fibers. The durability of the functional fabrics was verified by rubbing the sample thrice and then washing it. The color fatness to rubbing was observed using a fatness tester, and the results are shown in Figure 6c,f. Generally, the grade of the color fatness of the grafting fabrics from Figure 6c was obviously higher than that of Figure 6f after washing and drying three times, and the specific fatness values of the samples are shown in the corner tables. The grade of color fatness was close to level-3 in Figure 6f, which is consistent with the ordinary outwear clothing fabrics’ value based on the ISO105/A03−1993 and GB/T251-2008 standards [47]. The permanent and reliable interface grafting is a safeguard for the subsequent fastness problem in the wearing and washing process of the protective fabrics.

**Figure 7:**
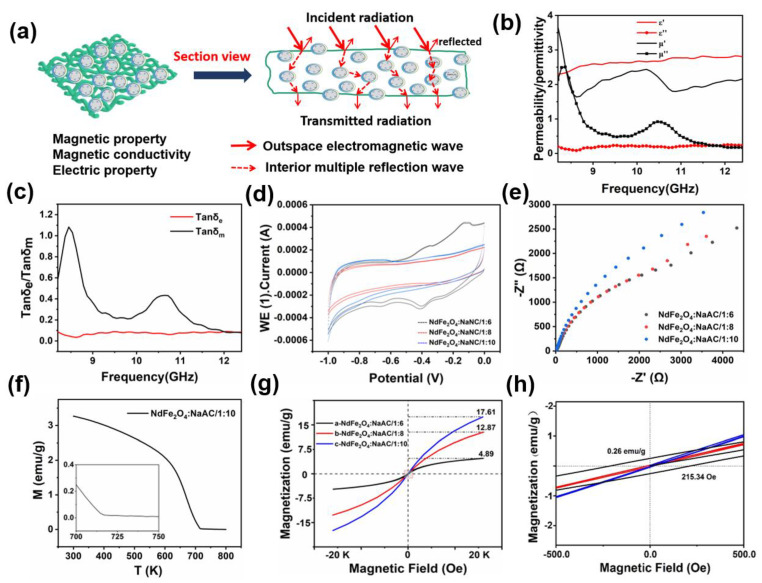
Illustration and electromagnetic properties of the functional fabrics. (**a**) The graphic mechanism of the functional fabrics; (**b**) electromagnetic parameters and (**c**) loss of the electromagnetic parameters; (**d**) CV curves and (**e**) double Ohm curves of the electrochemistry performance; (**f**) M−T curve, (**g**) M−H curves, and (**h**) enlarged M−H curves of the magnetic properties.

In general, when the prepared magnetic nanoparticles were grafted onto the surface of the cotton fabrics in a gradient, their small particles were evenly scattered. This process allows for their use in electric and magnetic property testing and analysis. When the test frequency of electromagnetic wave radiation was incident upon the surface of the functional fabric, its magnetic energy was partially reflected in the fabric’s interior into the air as heat energy. Furthermore, the second part was absorbed by the multiple reflections on the functional component through dielectric and magnetic loss in the interior of the fabrics [48]. Finally, the transmitted radiation was evidently attenuated to a low value for the electromagnetic protective materials, as shown in Figure 7a. Figure 7b presents the electromagnetic property parameters of the obtained sample under the frequency range of 8.2−14.2 GHz in the X band, including the real and imaginary part, and the dielectric and magnetic loss were also calculated [49]. In addition, the imaginary parts would be transferred to each other until the end of the loss by the functional body through dielectric and magnetic loss [50]. These losses are often verified based on several electromagnetic parameters, such as permeability and permittivity. Furthermore, the real permeability *ε’* and permittivity *µ’* are connected to the dispassion capacity of the test fabric, and the imaginary parts *µ’’* and *ε’’* are related to the degradation capability of the electromagnetic energy, respectively [51]. The dielectric loss (tanδ_e_ = *ε’’/ε’*) and magnetic loss (tanδ_m_ = *µ’’/µ’*) curves of the fabric were generated (Figure 7c). The *ε’* values were near 2.5, and the *µ’* values floated around 2. When the frequency was under 9 GHz, the *µ’* and *µ’’* values showed a downtrend, which could be attributed to the magnetic dipoles’ movement for the magnetic loss property of NdFe_2_O_4_ [52]. However, the *ε’’* and *µ’’* values were relatively low, indicating a difficulty in transferring the electric and magnetic field forces [53]. The as-prepared fabrics exhibited a weak conductive ability, as shown in Figure 7d,e, which could be attributed to the magnetite NdFe_2_O_4_ NPs’ low interior motion of the magnetic dipole in varying magnetic fields for transferring electric fields, thus supporting the results in Figure 7b,c [54]. In comparison with the existing samples, the fabrics with different NPs had little difference in their specific capacitance (*Cp*) values and dielectric constants. The best *Cp* value was 0.0551 *F/g*, which was recorded from the fabric with NPs at 1:6 NaAC, and this value could be calculated based on the area of the curve through the CV results in Figure 7d. Furthermore, Figure 7e exhibits a different dielectric capacity for the obtained samples with a slight discrepancy in the curve radian. The double Ohm curve for such fabric with NPs at 1:6 NaAC also showed the lowest curve radian for the best dielectric property, which was consistent with the results in Figure 7d [55,56].

The stability of the functional fabrics at high temperatures was evaluated based on their magnetic susceptibility under a changing temperature M−T curve and magnetizing M−H curve. The samples exhibited fine magnetic properties and magnetic loss performances as the temperature increased from 300 K to 800 K (Figure 7f), and the magnetization ability decreased sharply from 3.3 emu.g^−1^ to close to 0 emu.g^−1^, thus confirming the loss velocity of magnetic properties in hot environments [57]. Furthermore, the three as-prepared samples exhibited obvious differences in magnetic properties from Figure 7g and the enlarged picture Figure 7h. The fine magnetic value of the functional fabric with NdFe_2_O_4_ NPs at 1:10 NaAC was 17.61 emu.g^−1^, which was higher than 12.67 emu.g^−1^ for the sample with 1:8 NaAC and 4.89 emu.g^−1^ for the sample with 1:6 NaAC, which belonged to the reduction action of different mole ratios of NaAC in the synthesis process [58,59]. The as-prepared fabrics with NdFe_2_O_4_ NPs showed fine magnetic properties with weak dielectric properties that need to be improved, proving the effect of the obtained particle size on the electromagnetic properties for the finally functional protective fabrics.

## 3. Experiment

### 3.1. Materials

All the reagents, including ferric trichloride (FeCl_3_·6H_2_O), neodymium trichloride (NdCl_3_·6H_2_O), polyethylene glycol (PEG), ethylene glycol (EG), sodium acetate (CH_3_COONa, NaAC), glyceric acid (C_3_H_6_O_4_), N,N′−dissuccinimide carbonate (C_9_H_8_N_2_O_7_, DSC), and alkali (NaOH) were obtained from Aladdin Industrial and Sinopharm Reagent Corporation. Ultrapure water with a conductivity of 18.33 Ω/cm was prepared using a deionizing water purification system (PT−10T, Hitech Instruments Co., Ltd., Shanghai, China). Natural cotton yarn with a fitness of 2 × 32^S^ was provided by Haian Country Lianci Textile Co., Ltd. (Nantong, China).

### 3.2. Synthesis of Nd^3+^-doped Fe_3_O_4_ NPs

Spherical Fe_3_O_4_ NPs with uniform structures were prepared using a repeated synthesis method similar to the process described in our previous study [9]. Approximately 0.15 moles of NdCl_3_·6H_2_O were added to the FeCl_3_ solution, and the mixture was homogeneously dispersed and placed into a Teflon−lined container for the solvothermal reaction. Then, the NdFe_2_O_4_ NPs were obtained. The mole ratio of NaAC, a key reduction agent, was varied at different times: 1:6, 1:8, and 1:10 times as much as iron. Furthermore, a solvothermal reaction time of 12 h resulted in controllable sized NdFe_2_O_4_ NPs.

### 3.3. Preparation of Functional Fabrics with Modified NPs

First, plain weave fabrics with 2 × 32^S^ cotton yarns were finished through a proofing rapier loom (Y300S, Automatic Rapier Loom Machine, Nantong, China) at 240 yarns per 10 cm centimeters in both warp and weft directions. The cotton fabrics were then subjected to alkali treatment for the optimization of water absorption for the subsequent functional finishing. The alkali treatment was conducted at concentrations of 15%, 20%, 25%, and 30%. The best alkali treatment concentration on the cotton fabric was found to be 25%, resulting in 10.37% water absorption. The obtained NdFe_2_O_4_ NPs were modified using glyceric acid, allowing a connection with −OH on the cotton fabric through a bridging agent, DSC. The prepared samples were washed thrice and dried to remove the residual reagent.

### 3.4. Characterizations

The morphology of the NPs and functional fabrics were observed using a TM4000Plus scanning electron microscope (SEM, Hitachi, Japan) and a JEM−2100Plus transmission electron microscope (TEM, Jeol, Japan) connected with an energy dispersive X−ray (EDS, Jeol, Japan). X−ray powder diffraction (XRD, Bruker−D8, 10−70°, Germany), IS50 Fourier infrared spectroscopy (FT−IR, Nicolet 6700, America) with KBr pellets, dynamic light scattering (DLS, Vasco, Portugal), and X−ray photoelectron spectroscopy (XPS, Thenno, ESCALAB250, America) were carried out. The magnetic properties of the obtained nanoparticles and fabrics were analyzed using the Lake Shore 7307 vibrating sample magnetometer (VSM) from −20 KOe to 20 KOe at room temperature and a physical property measurement system (PPMS, Quantum Design PPMS 9) at 300−800 K. The color fatness to the rubbing of fabrics was detected using an instrument (YG5711−II, Meibon Instruments Co., Ltd., Quanzhou, China). The electrical properties of the fabrics were tested using an electrochemical working station measurement (PGSTAT302N, Metrohm Autolab, Kanaalweg, The Netherlands). The electromagnetic wave absorption performance of the samples was measured using an N5234A vector network analyzer (VNA, Agilent, Santa Clara, CA, America) based on a wave−guide method from 8.2 GHz to 12.4 GHz.

## 4. Conclusions

Novel Nd^3+^−doped Fe_3_O_4_ nanoparticles based on a microwave synthesis method and a surface modification were grafted onto cotton fabrics, and their magnetic protective properties were examined. The structural characterization of the obtained doping nanoparticles showed a spherical shape and a good dispersion for fine magnetic properties that can be attributed to the contribution of nano−structural spinel ferrite. In addition, the controllable particle size was confirmed for the electromagnetic properties of the obtained nanoparticles and final fabrics. The surface modification on NdFe_2_O_4_ nanoparticles and alkali treatment on cotton fabrics for rich hydrophilic groups were further determined using a bridging agent for durable interface bonding, and the samples showed fine color fatness to rubbing at level−3 after washing. The final structure and obvious hysteresis loop results of the obtained fabrics had a maximum magnetic saturation value of 17.61 emu.g^−1^ and weak electric properties for magnetic protective applications. However, the synthesis and surface modification method, which involved a grafting reaction, can also be applied for the preparation of other similar controllable nanoparticles for further functional fabric studies and product development, showing good universality.

## Data Availability

Not applicable.

## Sample Availability

Samples of the compounds mangetic protective fabrics are available from the authors.

## References

[1] Qi Y., Yin P., Zhang L., Wang J., Feng X., Wang K., Zhao L., Sun X., Dai J. (2019). Novel microwave absorber of Ni_x_Mn_1-x_Fe_2_O_4_/carbonized chaff (x = 0.3, 0.5 and 0.7) based on biomass. ACS Omega.

[2] Zhou B., Su M., Yang D.-X., Han G., Feng Y., Wang B., Ma J., Liu C., Shen C. (2020). Flexible MXene/siver nanowire-based transparent conductive film with electromagnetic interference shielding and electro-photo-thermal performance. ACS Appl. Mater. Interfaces.

[3] Zhan Y., Long Z., Wan X., Zhang J., He S., He Y. (2018). 3D carbon fiber mats/nano-Fe_3_O_4_ hybrid material with high electromagnetic shielding performance. Appl. Surf. Sci..

[4] Liang L.-Y., Li Q.-M., Yan X., Feng Y.-Z., Wang Y.-M., Zhang H.-B., Zhou X.-P., Liu C.-T., Shen C.-Y., Xie X.-L. (2021). Multifunctional magnetic Ti_3_C_2_T_x_ MXene/ graphene aerogel with superior electromagnetic wave absorption performance. ACS Nano.

[5] Lv H., Yang Z., Wang P.-L., Ji G., Song J., Zheng L., Zeng H., Xu Z.-J. (2018). A voltage-boosting strategy enabling a low-frequency, flexible electromagnetic wave absorption device. Adv. Mater..

[6] Zhang H., Guo Y., Zhang X., Wang X., Wang H., Shi C., He F. (2020). Enhanced shielding performance of layered carbon fiber composites filled with carbonyl iron and carbon nanotubes in the koch curve fractal method. Molecules.

[7] Singh A.-K., Shishkin A., Koppel T., Gupta N. (2018). A review of porous lightweight composite materials for electromagnetic interference shielding. Compos. Part B.

[8] Zhang X.-J., Zhu J.-Q., Yin P.-G., Guo A.-P., Huang A.-P., Guo L., Wang G.-S. (2018). Tunable high-performance microwave absorption of Co_1-x_S hollow spheres constructed by nanosheets within ultralow filler loading. Adv. Funct. Mater..

[9] Liu Q., Cao Q., Bi H., Liang C., Yuan K., Yang Y., Che R. (2016). CoNi@SiO_2_@TiO_2_ and CoNi@Air@TiO_2_ microspheres with strong wideband microwave absorption. Adv. Mater..

[10] Qiao M., Lei X., Ma Y., Tian L., He X., Su K., Zhang Q. (2018). Application of yolk-shell Fe_3_O_4_@N-doped carbon nanochains as highly effective microwave-absorption material. Nano Res..

[11] Xu J.-D., Li R.-S., Ji S.-R., Zhao B.-C., Cui T.-R., Tan X.-C., Gou G.-Y., Jian J.-M., Xu H.-K., Qiao Y.-C. (2021). Multifunctional graphene microstructures inspired by honeycomb for ultrahigh performance electromagnetic interference shielding and wearable application. ACS Nano.

[12] Song P., Liang C.-B., Wang L., Qiu H., Gu H.-B., Kong J., Gu J.-W. (2019). Obviously improved electromagnetic interference shielding performances for epoxy composites via constructing honeycomb structural reduced graphene oxide. Compos. Sci. Technol..

[13] Sridhar V., Lee I., Park H. (2021). Metal organic frameworks derived Fe-N-C nanostructures as high-performance electrodes for sodium ion batteries and electromagnetic interference (EMI) shielding. Molecules.

[14] Zhang X., Zhang S., Zhang K., Yan F., Zhu C., Yuan H., Zhang X., Chen Y. (2019). Interface-induced enhanced electromagnetic wave absorption property of metal-organic frameworks wrapped by graphene sheets. J. Alloys Compd..

[15] Giannakoudakis D.-A., Bandosz T.-J. (2019). Building MOF nanocomposites with oxidized graphitic carbon nitride nanospheres: The effect of framework geometry on the structural heterogeneity. Molecules.

[16] Song X.-L., Wu Y.-L., Zhang S.-R., Chen Z., Li Y.-G. (2020). NdFe_2_O_4_ nanoparticles: Synthesis, characterization and magnetic properties. Sci. Adv. Mater..

[17] Chen X., Shi T., Wu G., Lu Y. (2020). Design of molybdenum disulfide@polypyrrole compsite decorated with Fe_3_O_4_ and superior electromagnetic wave absorption performance. J. Colloid Interface Sci..

[18] Adam A., Parkhomenko K. (2021). Orienting the pore morphology of core-shell magnetic mesoporous silica with the sol-gel temperature. influence on MRI and magnetic hyperthermia properties. Molecules.

[19] Wei Q., Pei S., Qian X., Liu H., Liu Z., Zhang W., Zhou T., Zhang Z., Zhang X., Cheng H.-M. (2020). Superhigh electromagnetic interference shielding of ultrathin aligned pristine graphene nanosheets film. Adv. Mater..

[20] Ji B., Fan S.-W., Kou S. (2021). Microwave absorption properties of multilayer impedance gradient absorber consisting of Ti_3_C_2_TX MXene/polymer films. Carbon.

[21] Zirak M., Abdollahiyan A., Eftekhari S.-B., Saraei M. (2017). Carboxymethyl cellulose coated Fe_3_O_4_@SiO_2_ core-shell magnetic nanoparticles for methylene blue removal: Equilibrium, kinetic, and thermodynamic studies. Cellulose.

[22] Ebenezer C.N., Peter A.A. (2020). Multifunctional magnetic oxide nanoparticle (MNP) core-shell: Review of synthesis, structural studies and application for wastewater treatment. Molecules.

[23] Liu S., Mei J., Zhang C., Zhang J., Shi R. (2018). Synthesis and magnetic properties of shuriken-like nickel nanoparticles. J. Mater. Sci. Technol..

[24] Iqbal A., Symbyal P., Koo C.-M. (2020). 2D MXenes for electromagnetic shielding: A review. Adv. Func. Mater..

[25] Sankaran S., Deshmukh K., Ahamed M.-B., Pasha S.-K.-K. (2018). Recent advances in electromagnetic interference shielding properties of metal and carbon filler reinforced flexible polymer composites. Compos. Part A-Appl. S.

[26] Lei Z.-M., Tian D.-K., Liu X.-B., Wei J.-H., Rajavel K., Zhao T., Hu Y.-G. (2021). Electrically conductive gradient structure design of thermoplastic polyurethane composite foams for efficient electromagnetic interference shielding and ultra-low microwave reflectivity. Chem. Eng. J..

[27] Jia L.-C., Yan D.-X., Liu X.-F., Ma R.-J., Wu H.-Y., Li Z.-M. (2018). Highly efficient and reliable transparent electromagnetic interference shielding. ACS Appl. Mater. Interfaces.

[28] Xu Y., Yang Y., Yan D., Duan H., Zhao G., Liu Y. (2018). Gradient structure design of flexible waterborne polyurethane conductive films for ultraefficient electromagnetic shielding with low reflection characteristic. ACS Appl. Mater. Interface.

[29] Duan H., Zhu H., Gao J., Yan D., Dai K., Yang Y., Zhao G., Liu Y., Li Z. (2020). Asymmetric conductive polymer composite foam for absorption dominated ultra-efficient electromagnetic interference shielding with extremely low reflection characteristics. J. Mater. Chem. A.

[30] Abbasi H., Antunes M., Velasco J.-I. (2019). Recent advances in carbon-based polymer nanocomposites for electromagnetic interference shielding. Prog. Mater. Sci..

[31] Gupta S., Tai N. (2019). Carbon materials and their composites for electromagnetic interference shielding effectiveness in X-band. Carbon.

[32] Adebayo L.-L., Soleimani H., Yahya N., Abbas Z., Wahaab F.-A., Ayinla R.-T., Ali H. (2020). Recent advances in the development OF Fe_3_O_4_-BASED microwave absorbing materials. Ceram. Int..

[33] Mi H.-Y., Li H., Jing X., Zhang Q., Feng P.-Y., Ping H., Liu Y.-J. (2020). Superhydrophobic cellulose nanofibril/silica fiber/Fe_3_O_4_ nanocomposite aerogel for magnetically driven selective oil absorption. Cellulose.

[34] Luo X., Li H.-F., Deng D.-D., Zheng L., Wu Y.-B., Luo W.-J., Zhang M.-J., Gong R.-Z. (2022). Preparation and excellent electromagnetic absorption properties of dendritic structured Fe_3_O_4_@PANI composites. J. Alloys Compd..

[35] Xu L., Zhong W.-D., Yang C., Zhao R., Wu J., Li X., Yang N. (2022). Tailoring interfacial electron redistribution of Ni/Fe_3_O_4_ electrocatalysts for superior overall water splitting. J. Energy Chem..

[36] Bui D.-P., Nguyen T.-D., Vo T.-T.-L., Cao T.-M., You S.-J., Pham V.-V. (2021). SnO_2_-x nanoparticles decorated on graphitic carbon nitride as S-scheme photocatalysts for activation of peroxymonosulfate. ACS Appl. Nano Mater..

[37] Almessiere M.-A., Khan F.-A., Auwal I.-A., Sertkol M., Tashkandi N., Rehan I., Baykal A. (2022). Green synthesis, characterization and anti-cancer capability of Co_0.5_Ni_0.5_Nd_0.02_Fe_1.98_O_4_ nanocomposites. Arab. J. Chem..

[38] Sahar B.-K., Samuel R.-J., Sonia S., Stephen E.-H., Andrew D.-W. (2020). Structure-based virtual screening, synthesis and biological evaluation of potential FAK-FAT domain inhibitors for treatment of metastatic cancer. Molecules.

[39] Yang Y., Yang F., Wang H., Zhou B., Hao S. (2021). Amine-promoted Ru_1_/Fe_3_O_4_ encapsulated in hollow periodic mesoporous organosilica sphere as a highly selective and stable catalyst for aqueous levulinic acid hydrogenation. J. Colloid Interface Sci..

[40] Li P.-L., Zhang S., Zhu Y., Fan H., Ma W., Dong P., Wang W.-Z., Liu T. (2021). Polyimide-based graphene composite foams with hierarchical impedance gradient for efficient electromagnetic absorption. J. Mater. Chem. C.

[41] Sandhiya M., Subramani K., Sathish M. (2021). Augmenting the electrochemical performance of NiMn_2_O_4_ by doping of transition metal ions and compositing with rGO. J. Colloid Interface Sci..

[42] Geng L., Liu Q., Zhao J. (2022). In situ visualization of hierarchical agglomeration growth during electrochemical deposition of Cu nanocrystals in an open ionic liquid cell. Mater. Today Nano.

[43] Ma J., Wang T., Yu S., Zhang Y., Lyu B. (2020). Preparation and application of dialdehyde nanocellulose reinforced jatropha oil based polymer emulsions as leather fatliquors. Cellulose.

[44] Elshypany R., Selim H., Zakaria K., Houstafa A.-H. (2021). Magnetic ZnO crystal nanoparticle growth on reduced graphene oxide for enhanced photocatalytic performance under visible light irradiation. Molecules.

[45] Kuwa M., Harada M., Sato R., Teranishi T. (2020). Ligand-stabilized CoO and NiO nanoparticles for spintronic devices with antiferromagnetic insulators. ACS Appl. Nano Mater..

[46] Zeynizadeh B., Mohammadzadeh I., Shokri Z., Ali H.-S. (2017). Synthesis and characterization of NiFe_2_O_4_@Cu nanoparticles as a magnetically recoverable catalyst for reduction of nitroarenes to arylamines with NaBH4. J. Colloid Interface Sci..

[47] Joanna O., Jadwiga S.-L., Anetta W., Anna A., Katarzyna S.-C., Jakub Z., Teofil J. (2020). Antimicrobial activity and barrier properties against UV radiation of alkaline and enzymatically treated linen woven fabrics coated with inorganic hybrid material. Molecules.

[48] Ding K., Liu Z., Xiao C.-F. (2021). Fabrication of a novel one-step coating hyper-hydrophobic fluorine-free TiO_2_ decorated hollow composite membrane for use in longer-term VMD with enhanced flux, rejection, anti-wetting and anti-fouling performances. Nanoscale.

[49] Zhang Y., Fulajtárová K., Kub M., Mazur M., Shamzhy M., Hronec M. (2019). Controlling dispersion and accessibility of Pd nanoparticles via 2D-to-3D zeolite transformation for shape-selective catalysis: Pd@MWW case. Mater. Today Nano.

[50] Li G.-H., Ma S.-P. (2022). High-quality ferromagnet Fe_3_GeTe_2_ for high-efficiency electromagnetic wave absorption and shielding with wideband radar cross section reduction. ACS Nano.

[51] Sun S., Wang D., Feng Z., Tan W. (2020). Highly efficient unidirectional forward scattering induced by resonant interference in a metal-dielectric heterodimer. Nanoscale.

[52] Huang H., Gao Y., Fang C.-F., Wu A.-M., Dong X.-L., Kim B.-S., Byun J.-H., Zhang G.-F. (2018). Spray granulation of Fe and C nanoparticles and their impedance match for microwave absorption. J. Mater. Sci. Technol..

[53] Zhang C.-J., McKeon L., Kremer M.-P., Park S.-H., Ronan O., Seral-Ascaso A., Barwich S., Coileain C.-O., McEvoy N., Nerl H.-C. (2019). Additive-free MXene inks and direct printing of micro-supercapacitors. Nat. Commun..

[54] Xia S.-H., Wei C.-Y., Tang J.-C., Yan J.-H. (2021). Tensile stress-gated electromagnetic interference shielding fabrics with real-time adjustable shielding efficiency. ACS Sustainable Chem. Eng..

[55] Ye Y., Jiang Z., Zou Y., Chen H., Guo S., Yang Q., Chen L. (2020). Evaluation of the inhibition behavior of carbon dots on carbon steel in HCl and NaCl solutions. J. Mater. Sci. Technol..

[56] Qu Z., Wang Y., Yang P., Zheng W., Li N., Bai J., Zhang Y., Li K., Wang D. (2022). Enhanced electromagnetic wave absorption properties of ultrathin MnO_2_ nanosheet-decorated spherical flower-shaped carbonyl iron powder. Molecules.

[57] Yuan B.-G., Li J., Xia M.-M., Zhang Y., Lei R.-Y., Zhao P., Li X. (2021). Synthesis and electrochemical performance of hollow-structured NiO + Ni nanofibers wrapped by graphene as anodes for Li-ion batteries. Nanotechnology.

[58] Haider W.-A., Tahir M., He L., Mirza H.-A., Zhu R., Han Y., Mai L. (2020). Structural engineering and coupling of two-dimensional transition metal compounds for micro-supercapacitor electrodes. ACS Cent. Sci..

[59] Han M., Shuck C.-E., Rakhmanov R., Parchment D., Anasori B., Koo C.-M., Friedman G., Gogotsi Y. (2020). Beyond Ti_3_C_2_T_x_: MXenes for electromagnetic interference shielding. ACS Nano.

